# Preliminary Study on Growth and Yield Potential of Ten Elite Lines of Quinoa (*Chenopodium quinoa*) Cultivated under Varying Sowing Dates

**DOI:** 10.3390/plants11162116

**Published:** 2022-08-15

**Authors:** Ariba Asif, Shahbaz Khan, Bareera Asif, Danish Ibrar, Zuhair Hasnain, Muhammad Shoaib Ismail, Muhammad Rizwan, Sami Ullah, Saqib Bashir, Afroz Rais, Sohail Irshad, Tasawer Abbas, Naila Farooq, Jawaher Alkahtani, Mohamed S. Elshikh

**Affiliations:** 1 Department of Botany, University of Agriculture, Faisalabad 38040, Pakistan; 2 National Agricultural Research Centre, Islamabad 45500, Pakistan; 3 Department of Chemistry, Faculty of Chemical and Biological Sciences, Islamia University Bahawalpur, Bahawalpur 63100, Pakistan; 4 Department of Agronomy, PMA-Shah Arid Agriculture University, Rawalpindi 46000, Pakistan; 5 Department of Agronomy, MNS-University of Agriculture, Multan 64200, Pakistan; 6 Department of Agronomy, Gomal University, Dera Ismail Khan 29220, Pakistan; 7 Department of Soil & Environmental Science, Ghazi University, Dera Ghazi Khan 32200, Pakistan; 8 Department of Botany, Sardar Bahadur Khan Women’s University, Quetta 08763, Pakistan; 9 Inservice Agriculture Training Institute, Sargodha 40100, Pakistan; 10 Department of Soil & Environmental Sciences, College of Agriculture, University of Sargodha, Sargodha 40100, Pakistan; 11 Department of Botany and Microbiology, College of Science, King Saud University, Riyadh 11451, Saudi Arabia

**Keywords:** germplasm, growth, quinoa, sowing dates, yield

## Abstract

Optimization of agronomic practices for cultivation of quinoa, a super food and climate resilient crop, is critical to obtain its maximum grain yield under the current scenario of climate change. In the present experimentation, we evaluated the appropriate sowing time of ten elite lines of quinoa, already screened from USDA germplasm. Seeds of each line were sown in the experimental area at Square No. 22, Block No. 5, Directorate of Farm, University of Agriculture, Faisalabad, Pakistan on 15 November, 30 November and 15 December during quinoa cultivation season of 2019–2020. Sowing time significantly affected the emergence percentage, days taken to anthesis, chlorophyll contents, sodium and potassium concentrations in leaf, plant height, stem diameter, number of leaves and leaf area, panicle length, grain yield and 1000-grain weight. Lines; PIA-922, PIA-924, PIA-928 and PIA-929 performed better under first sowing and produced higher grain yield as compared to other lines. Similarly, PIA-921, PIA-922, PIA-925 and PIA-932 produced maximum biomass and grain yield under second sowing date while in case of third sowing date, PIA-926, PIA-928, PIA-930 and PIA-931 were observed more responsive regarding growth and yield attributes. A diversified pattern of agronomic, growth and yield contributing attributes of quinoa lines was observed when cultivated under varying sowing dates. The collected data will be very informative for the breeders and agronomists during selection and variety development process in future.

## 1. Introduction

Quinoa (*Chenopodium quinoa*) is a pseudo-cereal crop native to the South American Andean region which has increased its popularity worldwide. It was familiarized recently in Northern America, Africa, Asia and Europe. Quinoa is currently preferred as an alternate to conventional crops, despite its adaptability to marginal soils, drought, salinity and frost [1,2,3]. It contains carbohydrates (58–68%), proteins (10.4–17.0%) and fats (2–10%) making it a perfect energy source that is released gradually into the body due to its high fiber content (6.3%). Vitamins including A, B, C, E and minerals, i.e., Zn, Mg, Cu, Fe, Ca [4,5] are also present in its grains. Grains of quinoa are gluten free, and therefore used as supplement for the people with celiac disease [6,7]. Compared with other cereal crops, it has great nutritional features, due to which it can be included in a proper diet full of nutrients [4]. It is a valuable source of various nutrients, so is beneficial with many other food kinds to attain good overall diet. Keeping in view its importance, the United Nations announced the year 2013 as international year of quinoa [8]. Globally, demand for consumption is rising on a daily basis and an average of up to 2% edible grain production is estimated annually to ensure food safety [9,10].

Diversification in quinoa usually have variations in tissue phenology, morphology and chemical formation [11]. Nevertheless, photoperiod exposure is the most vital aspect in the emergence of new diversities recognized at larger latitudes. In most cases, quinoa is a short-day plant where the development of panicles in any photoperiod can be disrupted by seed filling and mellowing [12]. Sowing time is the most crucial activity of quinoa crop that has a great impact on the emergence, density level and biological yield of the crop. The emergence of seed depends upon the weather conditions of the region, line, moisture content of soil and the seeding depth. The sowing period in the southern Altiplano is from late August to early December, while the planting time is between October to November in the central and northern Altiplano, depend on rainfall and temperature [13]. Nonetheless, seasonal variations control factors such as temperature fluctuation and rainfall pattern, which can be overcome by monitoring the sowing time. Sajjad et al. [14] reported that quinoa crop sown on 15 January took more days to true leaf and bud formation. They also observed that early sowing performed better and showed good emergence and stand establishment than late sowing.

Shams and Galal [15] revealed that seeding of quinoa on 15 December resulted in maximum plant height, weight of the main head, 1000-grain weight and grain yield per plant under the climatic conditions of Egypt. Hirich et al. [16] explored the impacts of seeding date on quinoa growth throughout number of trials showed for alteration of quinoa. Seeding dates influenced the expansion and productivity. Maximum dry matter production and seed production were attained when quinoa seeded in November and at the start of December. Initial seeding in November to December produced better plant growth when temperature was low in January and February, and when damaging mildew was found in March. Awadalla and Morsy [17] studied the effect of planting dates and nitrogen levels on the plant height and number of branches per plant, dry weight per plant, weight of seeds per plant and biological yield. They concluded that planting at 1 November quinoa genotypes had the highest values of seed yield and protein content. Results depicted a strong correlation between the yield and its components with respect to sowing time. Planting of quinoa on 1 November resulted the highest values of economic return. The short growing season is a major constraint for growth in northern parts of Europe, Canada and high-altitude areas, as quinoa requires a maximum production time of 150 days in order to secure seed harvest [18]. Early maturity is therefore one of the most important features if quinoa is cultivated under these conditions. There is strong potential for increased quinoa production in southern Europe, in the United States, and in other parts of Africa and Asia. In Morocco, quinoa has been tested with varied yield according to sowing date, cultivar and soil under various climatic and soil conditions [19].

Nonetheless, Bolivia and Peru, the main producing countries of quinoa, cannot cope with the rising demand in the world. In Pakistan, 2009 was the year in which quinoa was familiarized to attain the upgrade production in numerous agro-climatic situations of the country [3,20]. Its farming time in Pakistan varies from the start of November to the end of December when all the climatic conditions are suitable for its sprouting [14]. It is also reported that the potential of the superior features of quinoa crop has not been completely discovered and utilized. Being a newly introduced crop in this region, it is necessary to explore the available germplasm regarding maximum yield linked with its adaptability in local agro-climatic conditions under varying sowing dates. Therefore, a field study was planned and conducted at the Research Farms Area, University of Agriculture to evaluate the influence of sowing dates on growth and yield of ten (10) elite lines of quinoa, previously screened from USDA germplasm and to optimize the sowing time for selected exotic quinoa lines under agro-climatic conditions of Faisalabad, Pakistan.

## 2. Results

**Figure 1:**
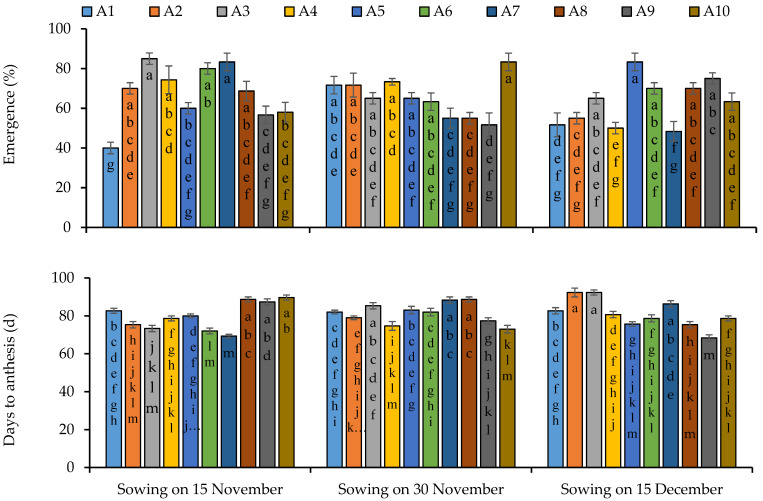
Emergence percentage and days to anthesis of quinoa lines grown under varying sowing dates. Bars sharing the same alphabet did not differ significantly at *p* < 0.05. Small alphabets (a, b, c. etc.) are used to express the statistically significant difference.

In the case of sowing on 15 November the maximum emergence percentage was found in lines 3 and 7, and line 2, 4, 6 and 8 were observed statistically similar in first sowing (Figure 1). Line 10 depicted maximum emergence percentage under sowing date of 30 November while lines 1, 2, 3, 4, 5 and 6 were recorded statistically similar. Regarding third sowing date on 15 December, line 5 was found on the top regarding emergence percentage and lines 6, 8, 9 and 10 performed statistically similar for emergence percentage (Figure 1). Sowing dates significantly influenced the days taken to anthesis as well as variations were also observed among the quinoa lines and their interactive impact was also noted statistically significant (Table S1). Lines 8, 9 and 10 took maximum to anthesis stage while minimum were observed in line 7 under first sowing date (Figure 1). Under second sowing date, maximum days taken to anthesis were found in lines 2, 7 and 8 while minimum in line 10. Lines 2 and 3 took the highest time period for anthesis stage when sowing was performed on 15 December while line 9 took least time (Figure 1).

**Figure 2:**
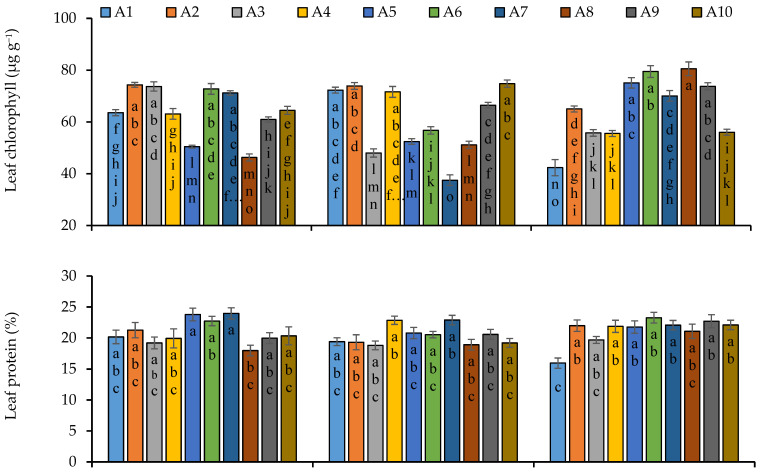

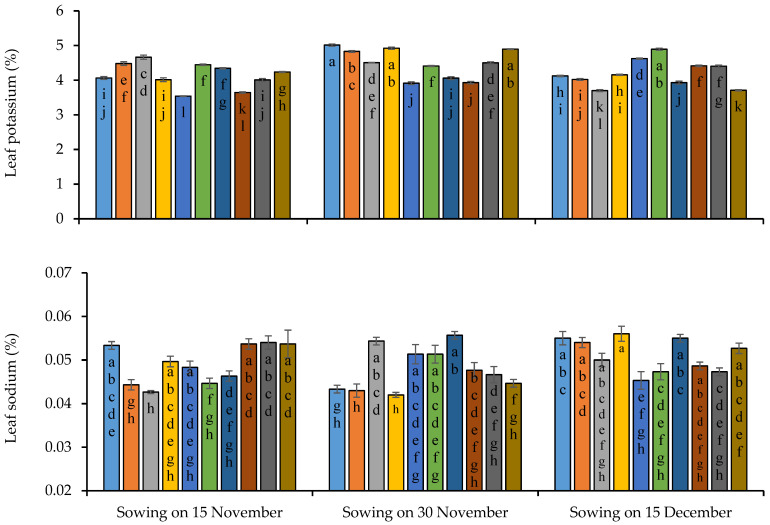
Leaf chlorophyll, leaf protein, leaf potassium and leaf sodium contents of quinoa lines grown under varying sowing dates at anthesis stage on fresh weight basis. Bars sharing the same alphabet did not differ significantly at *p* < 0.05. Small alphabets (a, b, c. etc.) are used to express the statistically significant difference.

Sowing time was observed statistically significant regarding the synthesis of chlorophyll pigments. Chlorophyll pigments were also found significant among the quinoa lines. The interactive effect of sowing times and lines was also recorded significant for chlorophyll pigments (Table S1). Lines 2, 3, 6 and 7 synthesized more chlorophyll pigments as compared to other lines while minimum pigments were observed in line 8 under sowing conditions of 15 November (Figure 2). Lines 1, 2, 4 and 10 were observed more responsive regarding the synthesize of chlorophyll pigments under sowing conditions of 30 November. Under the sowing conditions of 15 December, lines 5, 6, 8 and 9 synthesized maximum chlorophyll pigments. Protein content was not influenced by the sowing dates but was observed significant among the line and interaction was also statistically significant. Line 5 produced the highest protein content under first sowing date which was statistically similar with all other lines except line 8. In case of second sowing date, line 4 and 7 were recorded more prominent regarding the synthesis of protein content. All the lines performed better under third sowing date except line 1 regarding protein content. Leaf potassium (K) and sodium (Na) contents were significantly affected by sowing dates and even among the quinoa lines with significant interaction of sowing dates and lines (Table S1). Lines 1, 4, 5, 8, 9 and 10 under first sowing date, 3 and 7 under second sowing date and 1, 2, 3, 4, 7 and 10 under third sowing date accumulated more Na content in their leaves (Figure 2). Line 3 was comparatively more responsive regarding the accumulation of K contents in leaves under first sowing date. Under second sowing date, lines 1, 4 and 10 accumulated maximum K contents in their leaves while line 6 accumulated the highest K contents under third sowing date (Figure 2).

**Figure 3:**
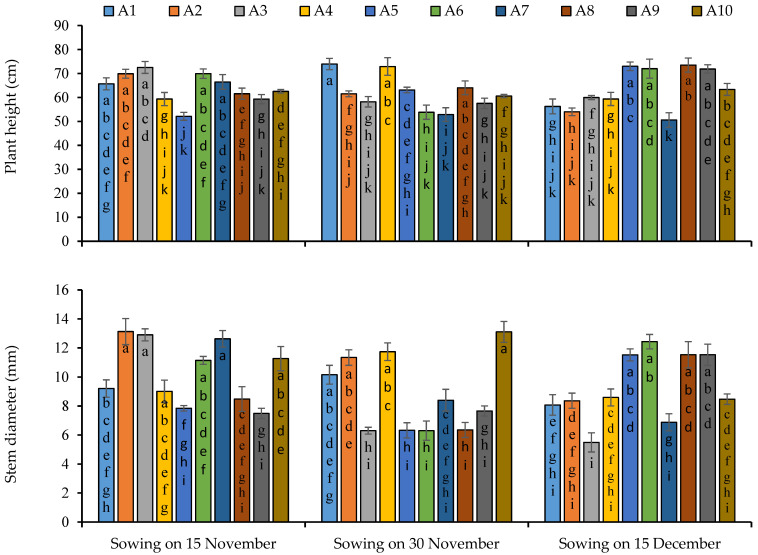
Plant height and stem diameter of quinoa lines grown under varying sowing dates. Bars sharing the same alphabet did not differ significantly at *p* < 0.05. Small alphabets (a, b, c. etc.) are used to express the statistical significant difference.

**Figure 4:**
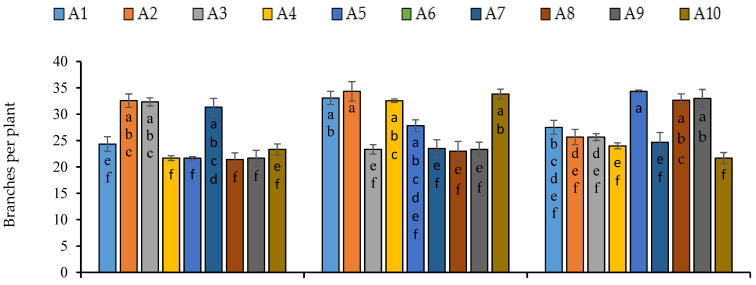

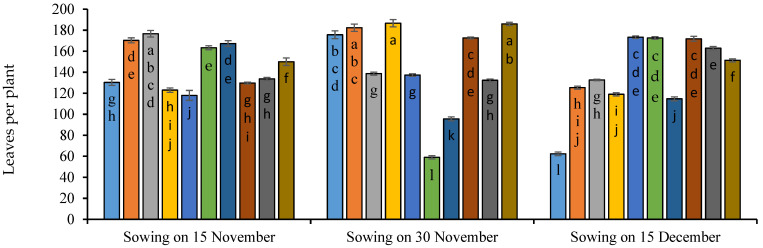
Number of branches and leaves per plant of quinoa lines grown under varying sowing dates. Bars sharing the same alphabet did not differ significantly at *p* < 0.05. Small alphabets (a, b, c. etc.) are used to express the statistically significant difference.

All the growth and yield attributes were significantly influenced by the sowing dates, except number of branches and biological yield, significant variations were observed among the lines regarding growth and yield attributes and interaction of sowing dates and lines was statistically significant (Table S1). Maximum plant height (63.92 cm), stem diameter (10.31 cm) and number of leaves per plant (146) were produced under first sowing while number of branches was not influenced by sowing dates (Figure 3). Regarding the performance of lines, maximum plant height (73.94 cm) was recorded in line 1 under second sowing date while line 7 produced the minimum height of 50.57 cm under third sowing date (Figure 3). Maximum diameter in lines 2 and 10, number of branches in line 1, 2 and 3, and number of leaves in lines 2, 8 and 10 were recorded (Figure 3). In case of interaction of sowing dates and lines, lines 3, 1, 5 produced maximum plant height under first, second and third sowing date respectively. Line 2 in first sowing date, 10 in second sowing date and 6 in third sowing date produced maximum stem diameter (Figure 3). Maximum number of branches was produced by the line 2 in first and second sowing, and 5 and 6 in third sowing. Maximum number of leaves was observed in line 3 under first sowing, in line 4 and 10 under seconding sowing, and lines 5 and 6 under third sowing (Figure 4).

**Figure 5:**
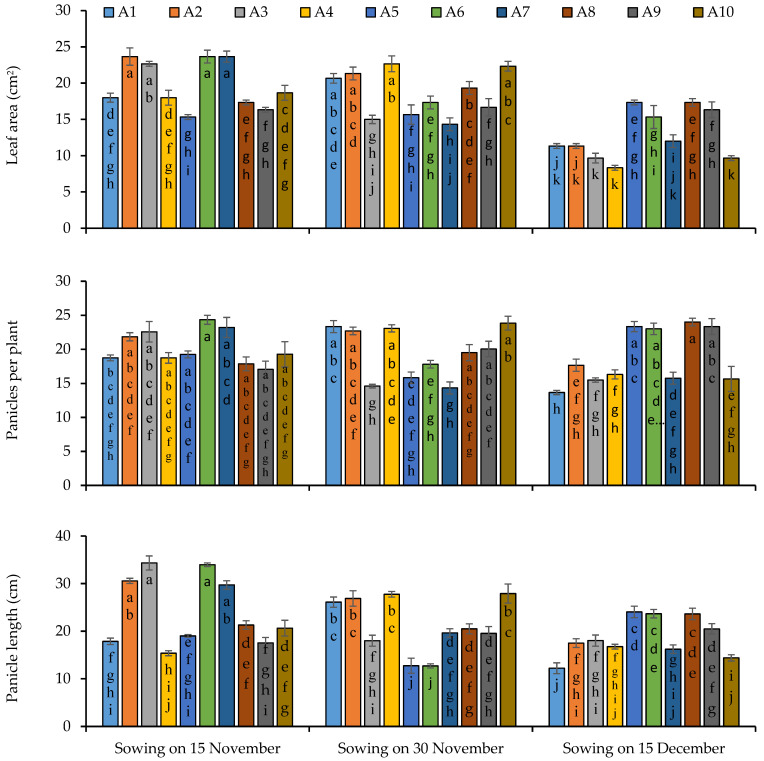
Leaf area (single leaf), number of panicles and main panicle length of quinoa lines grown under varying sowing dates. Bars sharing the same alphabet did not differ significantly at *p* < 0.05. Small alphabets (a, b, c. etc.) are used to express the statistically significant difference.

Sowing of 15 November was observed to be more responsive as compared to second and third sowing regarding leaf area, because maximum leaf area was observed under the first sowing while minimum was observed in the third sowing (Figure 5). Regarding genotypes’ performance, lines 2 and 6 produced the maximum leaf as compared to other lines and minimum leaf area was recorded in line 3. With respect to the interaction of sowing dates and lines’ performance, maximum leaf area was recorded in lines 2, 3, 6 and 7 under the first sowing, in lines 4 and 10 under the second sowing and lines 5 and 8 produced more leaf area under the third sowing (Figure 5). The maximum number of panicles was noted under the first sowing which was statistically similar to the second sowing followed by the third sowing. Line 6 produced the highest number of panicles while the minimum was observed in line 7 (Figure 5). Line 6 under the first sowing date and line 10 under second sowing date performed better regarding the number of panicles, and lines 8 under the third sowing (Figure 5). Maximum panicle length was recorded under the first sowing date while minimum was recorded under the third sowing, date and in case of lines, a maximum panicle length of 34.33 cm was produced by line 3 under the first sowing date and the minimum by line 1 under the third sowing date (Figure 5). Lines 3 and 6 were more prominent under the first sowing date regarding panicle length, while line 4 produced the smallest panicles of 15.37 cm under the same sowing date, and lines 1, 2, 4 and 10 produced longer panicles under second sowing date as compared to the other lines (Figure 5). Line 5 produced the longest panicles of 24.05 cm under the third sowing date while the minimum panicle length of 12. 22 cm was found in line 1 under the same sowing date (Figure 5).

**Figure 6:**
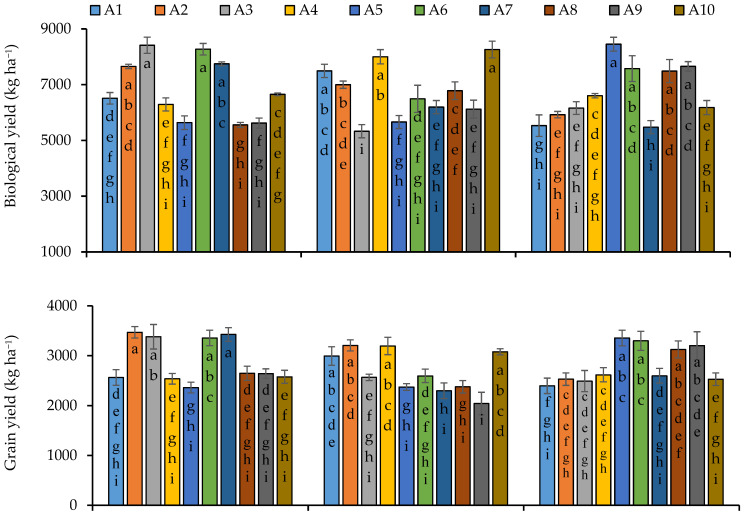

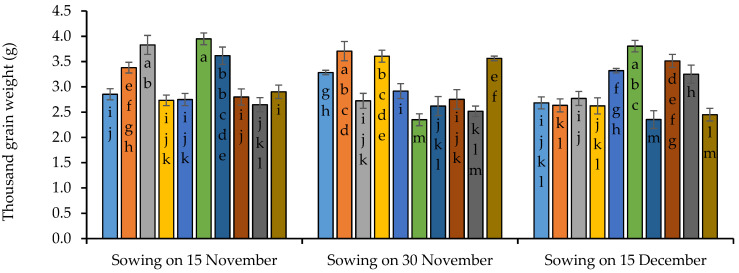
Biological yield, grain yield and thousand grain weight of quinoa lines grown under varying sowing dates. Bars sharing the same alphabet did not differ significantly at *p* < 0.05. Small alphabets (a, b, c. etc.) are used to express the statistically significant difference.

Biological yield was not statistically influenced by the sowing dates (Table S1); however, the first sowing was comparatively better for biological yield. Among the lines, line 6 produced the highest biological yield (7446.2 kg ha^−1^) followed by lines 10 (7029.9 kg ha^−1^) and 4 (6964.9 kg^−1^), respectively. In the case of the interactive effect of sowing dates and lines, line 3 and 6 under first sowing, lines 10 and 4 under second sowing and line 5 under the third sowing date produced more biological yield, respectively, as compared to other genotypes (Figure 6). Significant variations were recorded within the quinoa line regarding grain yield and 1000-grain weight, and even sowing dates significantly influenced these parameters. The interaction of sowing dates and quinoa line was noted as statistically significant for grain yield and 1000-grain weight (Table S1). Lines 2, 3 and 7 produced the maximum grain yield under the sowing conditions of November 15 and lines 1, 2, 4 and 10 under the second sowing date, and in case of the third sowing date of December 15, lines 5, 6, 8 and 9 were observed to be more responsive regarding grain yield (Figure 6). Overall, maximum grain yield was produced under the first sowing condition. In the case of lines’ performance, line 6 was on the top, producing a maximum grain yield of 3080 kg ha^−1^, which was statistically similar to lines 2, 3, 4 and 7 (Figure 6). Maximum 1000-grain weight was produced by line 6 (3.3689 g) followed by line 2 (3.240 g) (Figure 6). The sowing date of November 15 was observed to be more responsive regarding the 1000-grain weight as compared to the second and third sowing on 30 November and 15 December, respectively. With respect to sowing time, lines 3 and 6 in first sowing, 2 in the second sowing and 6 in the third sowing were found more responsive to produce the maximum 1000-grain weight (Figure 6).

**Figure 7:**
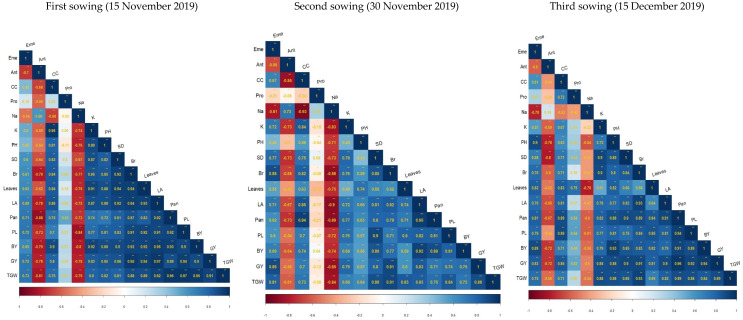
Pearson correlation among various biochemical, growth and yield attributes of ten quinoa lines grown under varying sowing dates. Eme = emergence percentage, Ant = days taken to anthesis, CC = chlorophyll content, Pro = protein, Na = sodium, K = potassium, PH = plant height, SD = stem diameter, Br = number of branches, LA = leaf area, Pan = number of panicles, PL = panicle length, BY = biological yield, GY = grain yield, TGW = thousand grain weight. * = significant at *p* < 0.05, ** = significant at *p* < 0.01.

Pearson correlation coefficients among all the studied morphological and physiological parameters of quinoa crops at three sowing dates are presented at Figure 7. At the first sowing date (15 November 2019), days to emergence depicted highly significant and positive correlation with all the parameters except for Na content and days to anthesis, wherein a negative correlation was observed. Meanwhile, days to anthesis depicted a negative and highly significant correlation with all other component traits, except for Na content, which showed a positive and significant correlation coefficient. This showed that as the days taken to anthesis increased, Na content in the leaves of quinoa plant also showed an upward elevation. Likewise, days to anthesis correlation coefficient values between Na content and all other component traits was negative and highly significant. Correlation values between chlorophyll content, potassium content, plant height, stem diameter, number of branches, number of leaves, leaf area, number of panicles, panicle length, biological yield, grain yield and thousand grain weight were found to be highly significant and positive. However, protein content of the quinoa crop revealed that proteins were not significantly correlated with any other component trait studied except for number of panicles and thousand grain weight, which showed a moderate (*p* < 0.05) and positive significance.

At the second sowing date (30 November 2019), days to emergence depicted a significant and positive correlation with potassium content (0.72 *), stem diameter (0.77 **), number of branches (0.88 **), leaf area (0.71 *), biological yield (0.69 *), grain yield (0.89 **) and thousand grain weight (0.81 **), while correlation coefficients for day to anthesis were found to be negative with all the studied traits, and most of coefficients values were also significant. Chlorophyll content showed a positive and significant association with all the physiological and morphological traits under observation except for sodium content (−0.93 **) which was negatively associated with chlorophyll content. However, protein content of quinoa crop revealed that proteins were not significantly correlated with any other component trait studied. Na content in quinoa plants also exhibited a strong and negative relationship with other plant characteristics, except for days to anthesis, which depicted that an increase in the Na content in the leaves of quinoa plant will eventually lead to early anthesis, which in turn results in reduction in other economically important traits such as seed yield and reduction in overall plant biomass. Correlation coefficient estimates between potassium content, plant height, stem diameter, number of branches, leaf area, number of leaves, panicles, panicle length, biological yield, grain yield and thousand grain weight were found to be strongly associated with each other in the positive direction at *p* < 0.01. It can be deduced from these correlation coefficient estimates that as the magnitude of one characteristic of quinoa plant rise, it helped to increase the magnitude of other above-mentioned traits as well.

During the third sowing date (15 December 2019), days to emergence depicted highly significant and positive correlation with all the parameters except for Na content (−0.78 **) and days to anthesis (−0.60 *), wherein a negative correlation was observed, while correlation coefficients for day to anthesis were found to be negative with all the studied traits, and most of the coefficients’ values are also significant. These results showed that as the days taken to anthesis increased, Na content in the leaves of quinoa plant also showed an upward elevation. Chlorophyll content showed a positive and significant association with all the physiological and morphological traits under observation except for Na content (−0.52 **), which is negatively associated with chlorophyll content. Protein content of quinoa plants that showed a non-significant correlation, in the first and second sowing date, depicted a positive and significant correlation in the third sowing date with most of the plants’ physiological and morphological attributes except for Na content (−0.42 **). As with days to anthesis, correlation coefficient values between Na content and all other component traits were negative and highly significant. Correlation values between chlorophyll content, potassium content, plant height, stem diameter, number of branches, number of leaves, leaf area, number of panicles, panicle length, biological yield, grain yield and thousand grain weight were found to be highly significant and in the positive direction. It can be deduced that due to the strong positive correlation between these traits, efforts to increase one trait will ultimately result in the increase in other economically important traits such as plant biomass and grain yield.

## 3. Discussion

Optimized production technology is one of the critical elements to obtain the maximum economic return of field crops. The current study was designed and conducted to examine the effects of various sowing dates on growth, productivity and yield of ten elite lines of quinoa. Our results revealed that there was significant difference for emergence, growth, physiological and biochemical attributes in quinoa under different sowing dates and between various lines. The results showed that the maximum emergence percentage was found under first sowing date (15 November) as compared to other sowing dates. The maximum emergence under first sowing may be due to the better optimal temperature requirements. The outcomes of current experimentation are supported by Bertero [9], who reported that temperature optima of the soil have a great impact on quinoa germination. These findings are consistent with already published data [21]. However, these findings were inconsistent with the results of Sajjad et al. [14], who reported that sowing dates and lines have non-significant influence on the germination characteristics of quinoa. In our case, results showed that lines had a strong impact on days to anthesis. These findings agree with Sajjad et al. [14], who reported that sowing dates significantly influence the growth duration of quinoa. The same findings were also reported in previous studies [18,21]. Similarly, according to Risi and Galway [22], sowing dates have a greater influence on the growth period of quinoa.

The chlorophyll content varied significantly between different lines as well as sowing dates. As to the individual effect of lines, the maximum chlorophyll content was recorded for those lines which have maximum emergence percentage as compared to other lines. The germination rates indirectly influence the chlorophyll content, as the space with canopy has influence on the leaves’ growth at lateral stage. Among the lines, greater variation among biochemical traits may be due to the genetic variability of each line [16]. For the sowing dates, similar findings were also reported by Choukr-Allah et al. [23]. The variation among the sowing dates regarding growth attributes may be due to the uplifted weather conditions for various sowing dates. As reported earlier, fluctuations in climatic conditions had a significant influence on quinoa growth. The genetic variability had a great impact on the performance of quinoa crop [16]. This evidence indicates that variation among growth characteristics for different lines may be due to genetic variability of various lines as well as climatic factors. In this study, treatments (sowing dates and lines) significantly influence the shoot length of quinoa. Among the sowing dates, the maximum increment in plant height was found in first sowing date. The findings of the current study regarding plant height are in line with the outcomes of Hirich et al. [16], who reported that growth characteristics varied significantly among lines mainly due to the genetic background of each line. The variation among the sowing date may be due to fluctuation in weather conditions. The varied climatic conditions for different sowing dates have impact on the growth traits of crops [24].

The results of the experimentation showed that sowing dates had a significant impact on the stem diameter, and there was a strong relation between lines and stem diameter. Fluctuations in climatic conditions significantly influence the growth parameters of quinoa. Variations in the optimum temperature either high or low cause severe reduction in growth traits. In addition, Bertero [9] reported that variations in growth traits were observed under different climatic conditions. The significant variation in stem diameter is mainly attributed to the genetic characteristics of specific lines. In addition to the stem diameter, the results of present study also revealed that sowing dates had a non-significant effect on number of branches per plant. However, lines showed a strong influence on number of branches per plant. These findings are in line with Mortensen and Gislerød [25], who reported that growth traits varied among various lines. In addition to the plant height, sowing dates and lines significantly influence the leaf area of quinoa. These findings are in line with the outcomes of Bertero et al. [11]. The authors reported that different photoperiod durations due to various sowing dates have a significant impact on the leaf surface area. In this study, the variations among lines may be due the genetic characteristics of each line as concluded by Hirich et al. [16].

In the present study, there was a strong variation for panicle length and number of panicles under different sowing dates and among the lines. In general, among sowing dates, maximum panicle length was recorded quinoa sown earlier as compared to other sowing dates. Additionally, lines also have significant impact on panicle length. Meanwhile, maximum number of panicles were reported for the first sowing. However, lowered panicle numbers were recorded under the third sowing date. In addition to sowing dates, lines also had significant impact on number of panicles. The maximum panicle length during early growing quinoa was mainly due to the optimal weather condition. The increase in temperature at lateral growth stages can caused a severe reduction in yield and yield attributes of quinoa [18]. Similar findings were also reported by Shah and Akmal [26], delay in sowing date caused a greater reduction in yield and yield attributes such as spike length. As compared to other parameters, sowing dates did not significantly influence the biological yield of quinoa. However, more increment in biological yield was found under early sowing of quinoa as compared to other sowing dates. These findings are inconsistent with results of Sajjad et al. [14], who found that late sowing significantly reduces the biological yield. In our case, maximum decrease was found under early sowing than other sowing dates. Similarly, our findings are also in line Nakano and Morita [27], who reported that early sowing produced more biomass than late sowing. In this study, lines depicted significant variation in economic yield of quinoa lines. The effects of sowing dates were also significant on economic yield. Among the lines, the maximum economic yield was reported for A-6 (PIA-928) compared to other lines of quinoa. Similar findings were reported by Sajjad et al. [14], who reported that lines were reported to have significant influence on the grain yield. Our findings are supported by Shah and Akram [26], who reported that early sown was responsible for the maximum production of grain yield in quinoa.

The thousand grain weight has a great contribution to determine the grain yield of crops. In our study, 1000-grain weight in quinoa significantly differed under different sowing dates and among the lines. Under different sowing dates, the maximum 1000-grain weight was recorded for early sowing quinoa as compared to other sowing dates. For quinoa lines, the maximum 1000-grain weight was recorded for A6 (PIA-928) compared to other lines. This was similar to findings by Sajjad et al. [14], who reported that early sowing quinoa (on 15 November) produced greater 1000-grain weight compared with late-sown quinoa. The greater 1000-grain weight may be due to fluctuation in weather conditions, as temperature showed a great influence on the yield [28]. Rezouki et al. [29] stated that agronomic practices, particularly sowing and harvesting periods, are crucial and play a significant role in yield production. Outcomes of presentation experimentation are in line with the findings of Akram et al. [30] as they reported that seed yield of quinoa lines was linked with physiological, morphological and phenological attributes. They also stated that some lines were found to have short duration while others had long duration. The window of plasticity for planting ranges from 15 October to 15 December, and favorable time for its growth and yield potential is during November under irrigated conditions [31,32]. Prolonged maturity and reduction in grain filling cause considerable decline in economical return and may be linked with a delay in planting time as well as specific response of lines.

## 4. Materials and Methods

### 4.1. Experimental Specifics

**Figure 8:**
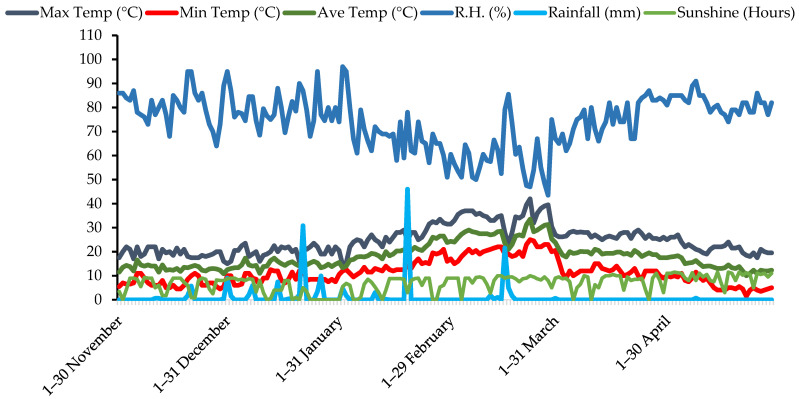
The weather data of experimental station from November 2019 to April 2020 during the growing season of quinoa crop.

The present study was designed to explore the cultivation and adaptation potential of quinoa germplasm under different sowing dates at agro-climatic conditions of Faisalabad, Punjab-Pakistan. The experiment was conducted during the quinoa growing season of 2019–2020 at Square No. 22, Block No. 5 having a size of 2.5 hectares, directorate of Farm, University of Agriculture, Faisalabad (30.35–31.47° N latitude and 72.08–73° E longitude), Pakistan. The experimental soil was sandy loam in texture having pH of 7.6, electrical conductivity at 0.69 dS m^−1^ and exchangeable sodium at 0.28 mmol_c_/100g. The concentrations of organic matter, total nitrogen, available phosphorus and potassium were 0.96%, 0.061%, 9.1 mg ka^−1^ and 211 mg kg^−1^ respectively. The weather conditions during the course of study are presented in Figure 8. From USDA germplasm, seeds of ten quinoa lines were collected from Alternate Crops Lab, University of Agriculture, Faisalabad, Pakistan for experimentation. The trial was executed in split plot arrangement under randomized complete block design keeping sowing dates in main plot and quinoa lines in sub plots having three replicates. Seeds of each line were sown in 75 cm wide lines by hand placement on flat surface using 8 kg ha^−1^ seed rate. The area an experimental unit was 112.5 m^2^ having ten rows and each row was 15 m in length. To fulfill the requirements of primary nutrients, murate of potash (60% K_2_O), diammonium phosphate (46% P_2_O_5_ and 18% N) and urea (46% N) were used. Furthermore, N, P and K were supplied at a rate of 75 kg ha^−1^, 60 kg ha^−1^ and 60 kg ha^−1^, respectively. Four irrigations were applied according to the requirement of crop. Hand weeding was performed twice throughout the course of experimentation to control the weed infestation. Disease attack was not observed, so chemical was not used is this regard.

### 4.2. Treatment Plan and Implementation

The following treatment plan was followed to study the above-mentioned objectives:

Factor A-Sowing dates:

First sowing: 15 November 2019.

Second sowing: 30 November 2019.

Third sowing: 15 December 2019.

During first sowing of quinoa on 15 November, the maximum, minimum and average temperature were 20 °C, 5 °C and 12.5 °C respectively. Relative humidity and rainfall were 68% and 0 mm respectively. On second sowing date, the maximum, minimum and average temperature were 16 °C, 5.5 °C and 10.8 °C, respectively, and relative humidity and rainfall were 89% and 0.4 mm respectively. In case of third sowing date, relative humidity and rainfall were recorded as 80% and 0 mm respectively while the maximum, minimum and average temperatures were observed as 20 °C, 12 °C and 16 °C respectively (Figure 8).

Factor B-Quinoa lines:

Total ten lines of quinoa were studied in the experimentation and their identities are given below.
A_1_ = PIA-921—ChileA_6_ = PIA-928—PeruA_2_ = PIA-922—Bolivia, La PazA_7_ = PIA-929—Chile, Bio-BioA_3_ = PIA-924—Bolivia, La PazA_8_ = PIA-930—Chile, Bio-BioA_4_ = PIA-925—Bolivia, PotosiA_9_ = PIA-931—United States, ColoradoA_5_ = PIA-926—PeruA_10_ = PIA-932—United States, New Mexico

### 4.3. Measurement of Emergence Percentage and Days Taken to Anthesis

The number of seeds was counted at the time of sowing and the emerged seeds were counted daily for the final emergence percentage. Final emergence percentage was calculated according to the follow formula;

Final emergence percentage = Final number of emerged seeds/Total number of seeds sown × 100

To record the days taken to anthesis, five plants were tagged in each experimental unit and examine daily and data were recorded and averaged.

### 4.4. Estimation of Leaf Chlorophyll, Protein and Mineral Contents

At anthesis stage, fully expanded leaves were harvest to collect the data regarding physiological and biochemical parameters. By the protocol of Arnon [33], chlorophyll *a* and *b* pigments were estimated. Leaf samples were soaked in 10 mL of 80% acetone solution for overnight period. Before running the leaf samples, 100% acetones were run as a control. Absorbance of the samples was taken on spectrophotometer at 645 and 663 nm. The following formulas were used for the estimation of chlorophyll pigments;

Chlorophyll *a* content = [12.7 (OD 663) − 2.69 (OD 645)] × V/1000 × W

Chlorophyll *b* content = [12.7 (OD 645) − 4.68 (OD 663)] × V/1000 × W

where V = volume of extract in mL; W = weight of fresh leaf sample in grams

Total chlorophyll contents were calculated by sum of chlorophyll *a* and *b* contents. The protein content was determined according to the procedure standardized by the Association of Official Analytical Chemistry [34]. The leaf sap was obtained by freezing the leaves collected from all treatments which were then ground and liquefied. The sap was then diluted for flame photometry. Flame photometer was used to find the Na^+^ content. Then, 10 mL of sap liquid was used to obtain a curve observing Na^+^ on the x-axis and the optical densities determined by flame photometer on the y-axis. The sodium content was then calculated by the help of a curve. To estimate the K^+^ contents, leaves samples were put into falcon tubes filled with 25 mL of 1% HNO_3_ solution for digestion on a hot plate at 85 °C for four hours. One milliliter was taken from the digested solution, and a volume of 10 mL was prepared to measure the K^+^ concentration in the leaf samples using a flame photometer (Model 360; Sherwood Scientific Ltd., Cambridge, UK).

### 4.5. Measurement of Growth and Yield Parameters

The crop was harvested manually with the help of a sickle when the stem and seed looked dried. Plants were harvested on 12 April 2020 based on the first sowing date, as they matured within 149 days. In cases of second and third sowing dates, plants were harvested on 17 April 2020 and 25 April 2020, respectively. After harvesting, plants were kept for one week in an open environment for sun drying. Five plants were selected from each experimental unit and the plant height and panicle lengths were measured with the help of a measuring scale and then averaged. Stem diameter was measured using a Vernier caliper from the bottom, mid and top of the stem from five randomly selected plants and then averaged. Five plants were randomly selected from every experimental unit and the numbers of branches, leaves panicle were counted manually and averaged. The leaf area of five randomly selected plants was calculated by using a graph method and an average was obtained. Each leaf was placed on the graph paper and the structure was marked, and the number of small boxes was counted and multiplied with the correction factor to measure the leaf area.

Biomass was harvested from 1 m^2^ from three different places within a treatment to record the biological yield. Digital electric balance was used to record the biological yield. Then, biological yield was converted in kg ha^−1^. Weight of grain for each treatment was recorded by a digital electric balance in kg to measure the grain yield and later expressed in kg per hectare (kg/ha^−1^). Grains were harvested by manual threshing and a thousand grains were counted and weighed using digital electric balance and averaged to measure the 1000-grain weight.

### 4.6. Statistical Analysis

Normal data distribution assumption was tested using SPSS v. 17.0 software 195 packages according to the Shapiro and Wilk method [35]. The combined ANOVA was carried out according to Snedecor and Cochran [36] to estimate the main effects of sowing dates and line and their interactions. F-test was used to test treatment significance at 5% probability level using “MSTAT-C” software package. Overall, means of the lines were compared using Tukey’s HSD Test at level of 5% possibility [37]. Pearson correlation was drawn among the biochemical, growth and yield attributes of quinoa lines.

## 5. Conclusions

There was a significant difference for emergence, growth, physiological and biochemical attributes in quinoa lines under different sowing dates. Among sowing dates, the better emergence, growth traits and physiological characteristics were found in early sowing of quinoa during mid-November. Among the quinoa lines, three groups were observed with respect to sowing time. In the case of early sowing (15 November), lines PIA-922, PIA-924, PIA-928 and PIA-929 performed better and produced higher grain yield. Lines PIA-921, PIA-922, PIA-925 and PIA-932 produced maximum biomass when cultivated on 30 November. Under late sown condition (15 December), PIA-926, PIA-928, PIA-930 and PIA-931 were found more responsive regarding growth and yield attributes.

## Data Availability

The data that support the findings of the current experimentation are available from the corresponding author upon reasonable request.

## Supplementary Materials

The following supporting information can be downloaded at: https://www.mdpi.com/article/10.3390/plants11162116/s1, Table S1: Analysis of variance (mean sum of squares) of emergence percentage, days to anthesis, chlorophyll, protein, sodium and potassium contents of leaf, plant height, stem diameter, number of branches and leaves, leaf area, number of panicles, main panicle length, biological and grain yield, and thousand grain weight.
