# Supplementary material for: Preliminary Study on Growth and Yield Potential of Ten Elite Lines of Quinoa (Chenopodium quinoa) Cultivated under Varying Sowing Dates
Source: Plants (Basel). 2022 Aug 15;11(16):2116. doi: 10.3390/plants11162116 (PMC9413402; doi:10.3390/plants11162116)
Supplement: Supplementary file 1 [file plants-11-02116-s001.zip › plants-1833168-supplementary.pdf]

Table S1. Analysis of variance (mean sum of squares) of emergence percentage, days to anthesis, chlorophyll, protein, sodium and potassium contents of leaf, plant height, stem diameter, number of branches and leaves, leaf area, number of panicles, main panicle length, biological and grain yield, and thousand grain weight.

SOV	DF	Emergence percentage	Days to anthesis	Chlorophyll content	Protein content	Sodium content	Potassium content	Plant height	Stem diameter
Sowing dates (SD)	2	158.7*	4.811*	158.47*	6.56NS	7.61^{-5}**	1.097**	35.075*	18.45*
Accessions (AC)	9	246.1**	57.82**	304.56**	19.02**	2.03^{-5}**	0.315**	65.423**	7.98**
SD × AC	18	527.9**	146.7**	373.07**	6.42*	7.76^{-5}**	0.528**	212.202**	21.34**

		Number of branches	Number of leaves	Leaf area	Number of panicles	Panicle length	Biological yield	Grain yield	Thousand grain weight
Sowing dates (SD)	2	53.98NS	611**	403.51**	15.94*	214.03**	142540NS	384319*	0.334**
Accessions (AC)	9	27.26**	1735**	10.15**	15.41**	10.92**	1172410**	227528**	0.244**
SD × AC	18	90.36**	4478**	43.45**	47.09**	150.61**	4226349**	661319**	0.984**

SOV = Source of variance, DF = Degree of freedom, NS = Statistically non-significant, * = significant at $P < 0.05$, ** = significant at $P < 0.01$.
